# The association between distal symmetric polyneuropathy in diabetes with all-cause mortality – a meta-analysis

**DOI:** 10.3389/fendo.2023.1079009

**Published:** 2023-02-16

**Authors:** Orsolya E. Vági, Márk M. Svébis, Beatrix A. Domján, Anna E. Körei, Solomon Tesfaye, Viktor J. Horváth, Péter Kempler, Ádám Gy. Tabák

**Affiliations:** ^1^ Department of Internal Medicine and Oncology, Semmelweis University Faculty of Medicine, Budapest, Hungary; ^2^ School of PhD studies, Semmelweis University Faculty of Medicine, Budapest, Hungary; ^3^ Department of Public Health, Semmelweis University Faculty of Medicine, Budapest, Hungary; ^4^ Diabetes Research Unit, Royal Hallamshire Hospital, University of Sheffield, Sheffield, United Kingdom; ^5^ Department of Epidemiology and Public Health, University College London, London, United Kingdom

**Keywords:** meta-analysis, cohort studies, all-cause mortality, diabetes mellitus, type 1 diabetes, type 2 diabetes, distal symmetric polyneuropathy

## Abstract

**Background:**

Distal symmetric polyneuropathy (DSPN) is a common microvascular complication of both type 1 and 2 diabetes with substantial morbidity burden and reduced quality of life. Its association with mortality is equivocal.

**Purpose:**

To describe the association between DSPN and all-cause mortality in people with diabetes and further stratify by the type of diabetes based on a meta-analysis of published observational studies.

**Data Sources:**

We searched Medline from inception to May 2021.

**Study Selection:**

Original data were collected from case-control and cohort studies that reported on diabetes and DSPN status at baseline and all-cause mortality during follow-up.

**Data Extraction:**

was completed by diabetes specialists with clinical experience in neuropathy assessment.

**Data Synthesis:**

Data was synthesized using random-effects meta-analysis. The difference between type 1 and 2 diabetes was investigated using meta-regression.

**Results:**

A total of 31 cohorts (n=155,934 participants, median 27.4% with DSPN at baseline, all-cause mortality 12.3%) were included. Diabetes patients with DSPN had an almost twofold mortality (HR: 1.96, 95%CI: 1.68-2.27, I2 = 91.7%), I^2 =^ 91.7%) compared to those without DSPN that was partly explained by baseline risk factors (adjusted HR: 1.60, 95%CI: 1.37-1.87, I^2 =^ 78.86%). The association was stronger in type 1 compared to type 2 diabetes (HR: 2.22, 95%CI: 1.43-3.45). Findings were robust in sensitivity analyses without significant publication bias.

**Limitations:**

Not all papers reported multiple adjusted estimates. The definition of DSPN was heterogeneous.

**Conclusions:**

DSPN is associated with an almost twofold risk of death. If this association is causal, targeted therapy for DSPN could improve life expectancy of diabetic patients.

## Introduction

The prevalence of diabetes mellitus is increasing rapidly worldwide: it is expected to reach 578 million in 2030 (1). Meta-analyses of prospective studies (mostly from high-income countries) have found that diabetes is associated with an almost two-fold risk of a wide range of vascular diseases. This increase was clearly present in both men and women and was independent of other major conventional vascular risk factors (2). In addition to the values of individual risk factors, the overall burden (estimated as the number of abnormal values) of risk factors showed a dose-response relationship with cardiovascular risk (3). Although diabetes patients with well-controlled conventional vascular risk factors have a comparable mortality risk to that of the background population (4), not all excess vascular and mortality risk can be explained by conventional vascular risk factors (such as smoking, hyperlipidemia, hypertension, etc.) (2).

Diabetic microvascular complications could be partially responsible for this unexplained vascular and mortality burden. Indeed, there is strong evidence from a meta-analysis of observational studies that decreased estimated glomerular filtration rate and urinary albumin to creatinine ratio are both associated with an up to 2 times increased mortality (5). Furthermore, a former meta-analysis of observational studies of diabetes patients reported a substantially increased risk of mortality among diabetic patients with cardiovascular autonomic neuropathy (CAN) (6, 7). In addition to CAN, distal symmetric polyneuropathy (DSPN) is also a prevalent chronic microvascular complication of both type 1 and type 2 diabetes (8). Although DSPN is most prevalent in diabetic persons, it was found in people with normoglycemia or prediabetes (9–12). As DSPN may start early in the course of metabolic alteration, it could be a burden throughout the whole spectrum of impaired glucose metabolism leaving these patients prone to its potential effects on mortality and morbidity.

Given the equivocal data on the association of DSPN with all-cause mortality, we aimed to conduct a systematic review and meta-analysis on the impact of DSPN on all-cause mortality. Our hypotheses were that (1) DSPN is related to all-cause mortality in both types of diabetes. Furthermore, we thought that (2) this relationship would be stronger in type 1 diabetes given the younger age at the diagnosis of these patients.

## Methods

### Data sources and search strategy

The search strategy was designed by a diabetes specialist with experience in diabetes epidemiology (AGT) with input from all other investigators in accordance with the Meta-analysis of Observational Studies in Epidemiology (MOOSE) guideline (13). MEDLINE databases from their inception until 31/MAY/2021 were searched. Controlled vocabulary supplemented with keywords was used to search for studies fulfilling the criteria of reporting on both sensory diabetic neuropathy and mortality. We imposed no limitation on regional origin or language of any findings. We used the following search phrase: ((senso* OR DSPN OR neuropat* OR “diabetic foot” OR (foot AND ulcer*) OR Charcot) AND (diabete* OR diabeti*) AND (mortality OR death)). We aimed to collect case-control and cohort studies of diabetic people where baseline DSPN status was reported and follow-up mortality by DSPN status could be retrieved. All studies had to be of original data. We did not impose restriction on the length of follow-up.

### Data extraction and quality assessment

Several approaches (including questionnaires, symptoms, physical examination, and equipment-based techniques) were accepted for the diagnosis of DSPN (**Table 1**) as long as the same validated approach was used consistently within a report. Quality assessment of the papers was completed by the Newcastle-Ottawa Scale (NOS). We translated the NOS scores into the following strata using the Agency for Healthcare Research and Quality (AHQR) standards (1): good quality: 3 or 4 stars in selection domain AND 1 or 2 stars in comparability domain AND 2 or 3 stars in outcome/exposure domain (2); fair quality: 2 stars in selection domain AND 1 or 2 stars in comparability domain AND 2 or 3 stars in outcome/exposure domain (3); poor quality: 0 or 1 star in selection domain OR 0 stars in comparability domain OR 0 or 1 stars in outcome/exposure domain (41). The corresponding authors of potentially eligible primary studies without the required information within their publication were not invited to contribute with their raw data.

**Table 1:** Baseline characteristics of observational studies included in the meta-analysis on the association between DSPN and all-cause mortality.

ID	Author (year)	# of centers	Level of care	Setting	T1DM/T2DM	n	% male	% T1DM	Age	DM duration	Follow-up	Definition of neuropathy	Med. record	Symp-toms	Question-naire	Physical exam.	Equipment	NOS
1	Bjerg L - ADDITION (2021) (14)	Multiple	Population-based	ADDITION cohort	no/yes	1445	58%	0%	60.9 ± 7.3	0	11.4	MNSI			yes			good
1	Bjerg L - DD2 (2021) (14)	Multiple	Population-based	DD2 cohort	no/yes	5028	58%	0%	65.5 ± 11.1	4.5 ± 1.7	2.2	MNSI			yes			good
2	Bjerg L (2019) (15)	Single	Tertiary	Steno cohort	yes/no	3828	54%	100%	45.1 ± 16.6	22.8 ± 16,6*	7.0	VPT					yes	poor
3	Brownrigg JRW (2014) (16)	Multiple	Primary	General UK population	no/yes	13043	52%	0%	63.8 ± 12.8	NR	2.5	Physical examination (10g monofilament)				yes		good
4	Cusick M - T1DM (2005) (17)	Multiple		RCT of people with retinopathy (ETDRS)	yes/no	1444	58%	100%	33.7 ± 10.3	18.2 ± 6.3	5.0	Physical examination (tuning fork)				yes		good
4	Cusick M - T2DM (2005) (17)	Multiple		RCT of people with retinopathy (ETDRS)	no/yes	2267	34%	0%	55.5 ± 8.3	13.8 ± 6.5	5.0	Physical examination (tuning fork)				yes		good
5	Forsblom CM (1998) (18)		Population-based	Diabetes register based cohort	no/yes	131	51%	0%	57.6 ± 0.6	9.2 ± 0.6	9.0	Physical examination, NCV				yes	yes	good
6	Foryoung (2018) (19)	Single	Tertiary	Retrospective cohort from Sub-Saharan Africa	no/yes	628	56%	0%	56.5 ± 10.5	3.6 ± 0.36	3.1	Hospital records (no definition)	yes					poor
7	Garofolo (2019) (20)	Single	Tertiary	Retrospective cohort of diabetic foot	yes/no	774	53%	100%	40.2 ± 11.7	19.4 ± 12.2	10.8	MNSI, physical examination (tuning fork, monofilament)			yes	yes		poor
8	Gregory R (1994) (21)	Single	Tertiary	Newly diagnosed diabetes cohort	no/yes	136	50%	0%	68 ± 10.5	NR	5.0	Physical examination (pinprick test)				yes		poor
9	Hansen (2021) (22)	Single	Tertiary	Prospective cohort	yes/no	946	51%	100%	48.4 ± 14.4	25 ± 4.3	6.0	VPT					yes	poor
10	Hicks (2021) (12)		Population-based	Prospective cohort	yes/yes	1195	53%	NR	61.4 ± 0.7	11.3 ± 0.6	13.0	Physical examination (10g monofilament)				yes		good
11	Hsu WC (2012) (23)		Population-based	Neuropathy screening cohort	no/yes	326	33%	0%	63.5 ± 9.5	6.6 ± 7	5.2	NCV					yes	good
12	Kaze (2021) (24)	Multiple		Prospective cohort (Look AHEAD)	no/yes	4098	38%	0%	58.3 ± 6.6	5 ± 5.2	9.5	MNSI			yes			good
13	Kloecker (2021) (25)	Multiple		Prospective cohort (ACCORD, ACCORDION)	no/yes	9405	63%	0%	62.8 ± 6.7	10 ± 7.4	7.7	MNSI			yes			poor
14	Kristensen SL (2018) (26)	Multiple		RCT of heart failure patients	yes/yes	964	78%	NR	61.4 ± 10.4	NR	1.9	Single question	yes					good
15	Lapin (2020) (27)	Single	Tertiary	Prospective cohort	no/yes	43945	48%	0%	64.6 ± 14	0	3.1	Electronic health record	yes					poor
16	Lester FT (1992) (28)	Single	Tertiary	Prospective cohort	yes/no	275	58%	100%	20.8 ± 11.3	7.1 ± 5.6	15.0	Symptoms, decreased sensation		yes		yes		poor
17	McEwen N (2016) (29)	Multiple	HMO	TRIAD cohort	yes/yes	6992	46%	5%	61 ± 13	12 ± 10	10.0	Medical records (no definition)	yes					poor
18	Navarro X (1996) (30)	Single	Transplant centre	Considered for transplant	yes/no	545	45%	100%	33.4 ± 9	19.4 ± 8.8	11.5	NCV					yes	poor
19	O’Brien IA (1991) (31)	Single	Tertiary	Cohort	yes/no	506	58%	100%	45 ± 18	15 ± 10	5.0	Physical examination, VPT				yes	yes	good
20	Scain SF (2018) (32)	Single	Tertiary	Retrospective cohort of diabetic foot	no/yes	918	47%	0%	62.4 ± 10.4	10.8 ± 8.1	12.0	Physical examination (10g monofilament)				yes		good
21	Seferovic JP (2018) (33)	Multiple		RCT of aliskiren	no/yes	8463	68%	0%	64.5 ± 9.7	82% >5 years	2.7	MNSI			yes			fair
22	Soedamah-Muthu SS (2008) (34)	Multiple	Tertiary	EURODIAB cohort	yes/no	2787	51%	100%	32.3 ± 10	14.3 ± 9.1	7.8	Symptoms, physical examination, VPT		yes		yes	yes	poor
23	Suarez GA (2005) (35)		Population-based	Rochester Diabetic Neuropathy Study	yes/yes	462	49%	33%	61.2 ± 15.7	18 ± 10.1	15.0	NIS, NSC, NCV, other quantitative sensation studies			yes		yes	poor
24	Sudore RL (2012) (36)		HMO	Cohort with high drop-out	no/yes	13171	49%	0%	60 ± 9.9	9.7 ± 8.2	2.0	Symptoms (non-standard questionnaire)		yes				poor
25	Vagi OE - T1DM (2021) (37)	Single	Tertiary	Retrospective cohort	yes/no	131	53%	100%	46 ± 12	13 ± 10	9.0	CPT					yes	good
25	Vagi OE - T2DM (2021) (37)	Single	Tertiary	Retrospective cohort	no/yes	1011	44%	0%	64 ± 10	7 ± 8	8.0	CPT					yes	good
26	Weis U (2001) (38)	Single	Tertiary	Prospective cohort	yes/no	147	56%	100%	32.3 ± 11.9	16.8 ± 9.2	14.0	Physical examination				yes		poor
27	Yokomichi (2021) (39)	Multiple	Tertiary	Japanese hospital-based cohort	no/yes	30834	64%	0%	64.4 ± 11.1	8.4 ± 8.3	7.5	Medical records (no definition)	yes					good
28	Ziegler D (2015) (40)	Single	Tertiary	Inpatient cohort	yes/yes	89	54%	32%	54 ± 14	11.5 ± 9.7	5.8	Symptoms, NSS, NIS, physical examination, NCV, VPT, TDT		yes	yes	yes	yes	poor

*Age at diagnosis.

Age, DM duration, Follow-up reported in years.

Mean±SD.

Questionnaires: MNSI, Michigan Neuropathy Screening Instrument; NIS, Neuropathy Impairment Score; NSC, Neuropathy Symptoms and Change Score; NSS, Neuropathy Symptom Score.

Equipment-based methods: NCV, Nerve Conduction Velocity; TDT, Thermal Discrimination Threshold; VPT, Vibration Perception Threshold; CPT, Current Perception Threshold.

ACCORD, Action to Control Cardiovascular Risk in Diabetes trial; ACCORDION, ACCORD Follow-On study; ADDITION, Anglo-Danish-Dutch Study of Intensive Treatment in People With Screen-Detected Diabetes in Primary Care; CPT, Current Perception Threshold. DD2, Danish Centre for Strategic Research in Type 2 Diabetes; DM, diabetes mellitus; ETDRS, Early Treatment Diabetic Retinopathy Study; HMO, Health Maintenance Organization; Look AHEAD, Look AHEAD (Action for Health in Diabetes) study; Med. Record, medical record; MNSI, Michigan Neuropathy Screening Instrument; NCV, Nerve Conduction Velocity; NIS, Neuropathy Impairment Score; NOS, Newcastle-Ottawa Scale; NR, not reported; NSC, Neuropathy Symptoms and Change Score; NSS, Neuropathy Symptom Score; T1DM, type 1 diabetes mellitus; T2DM, type 2 diabetes mellitus; TDT, Thermal Discrimination Threshold; TRIAD, Translating Research Into Action for Diabetes study; VPT, Vibration Perception Threshold;

Reviewers worked independently and checked all abstracts and selected full-text manuscripts for eligibility. Preselected manuscripts were evaluated by 2 independent reviewers for eligibility. The total number of included and excluded articles was documented by the reviewers, including reasons for exclusion or non-eligibility. Any disagreements were discussed with the study designer (AGT). Whenever reviewers disagreed and no consensus was found, the article was included into the full-text phase. Disagreements at full-text screening were resolved by consensus of all authors.

For all studies, we extracted information on study design, number and characteristics of participants (i.e. age, sex, type of diabetes), prevalence of DSPN, method of DSPN assessment, duration of follow-up, all-cause mortality, matching, and confounding factors. Additionally, in the case of multiple publications, we included the most up-to-date or comprehensive information.

From the given publications, we sought data on the association between DSPN and all-cause mortality with their respective 95% confidence intervals (CI) based on unadjusted (minimally adjusted) and fully adjusted models. For the unadjusted models, we collected raw numbers, odds ratios (OR), hazard ratios (HR), or incidence rate ratios (IRR) that were either unadjusted or adjusted for only age and sex. If any other variable (i.e. diabetes type, BMI, and diabetes duration, co-morbidities) was taken into account, the estimate was considered to be fully adjusted. If more than one model was reported, the one with the most co-variables was selected. At this stage, we excluded unadjusted estimates from those studies, where the population was referred specifically for the examination of sensory or autonomic neuropathy, given the high risk of collider bias in this setting. If a given paper reported adjusted estimates, those were used in the analysis.

### Data synthesis and data analysis

The outcome measure of this meta-analysis was pooled all-cause mortality among individuals with DSPN compared to those without DSPN. We pooled estimates with their 95% confidence intervals (CIs) using random effect meta-analysis, as this methodology works well even when heterogeneity between studies is substantial (42). We also pre-planned to run analyses stratified by the type of diabetes (type 1 diabetes, type 2 diabetes, or undefined/mixture of type 1 and type 2 diabetes). We also tested formally for a difference between the estimates for type 1 and type 2 diabetes using meta-regression. For the main analysis, we included all cohorts that provided either unadjusted or adjusted estimates (using the unadjusted estimates if available).

To assess statistical heterogeneity, visual inspection of the forest plots was used, followed by formal testing using the I^2^-statistic. This provides an estimate of the percentage of variability across studies due to heterogeneity rather than chance: I^2^ < 40% may represent low heterogeneity; 30-60% may represent moderate heterogeneity; 50-90% represent substantial heterogeneity; and 75-100% represent considerable heterogeneity (43). Publication bias was evaluated by visual inspection of the funnel plots and formally by Egger’s tests. Additionally, we tested for influence of individual studies using a meta-analysis influence test that eliminated included studies one by one.

Given the expected heterogeneity of the eligible studies, pre-planned sensitivity analyses (stratified by type of diabetes) were also carried out by restricting the analysis to (1) studies that adjusted for potential predictors of mortality (other than age and sex) (2), population-based investigations (3), studies that used semi-quantitative (physical examination including monofilament or tuning fork or pinprick tests) or quantitative (vibration or current perception threshold or nerve conduction test) methods for DSPN assessment, and to studies that were deemed to be of good quality according to the Newcastle-Ottawa Scale (41). A further sensitivity analysis was done using meta-regression with adjustment for diabetes duration to investigate whether a risk difference between type 1 and type 2 diabetes would be explained by different length of diabetes duration.

All statistical tests were two sided and used a significance level of p<0.05. We used STATA 15.1 (StataCorp, CollegeStation, TX) for all statistical analyses.

## Results

### Study selection

**Figure 1:**
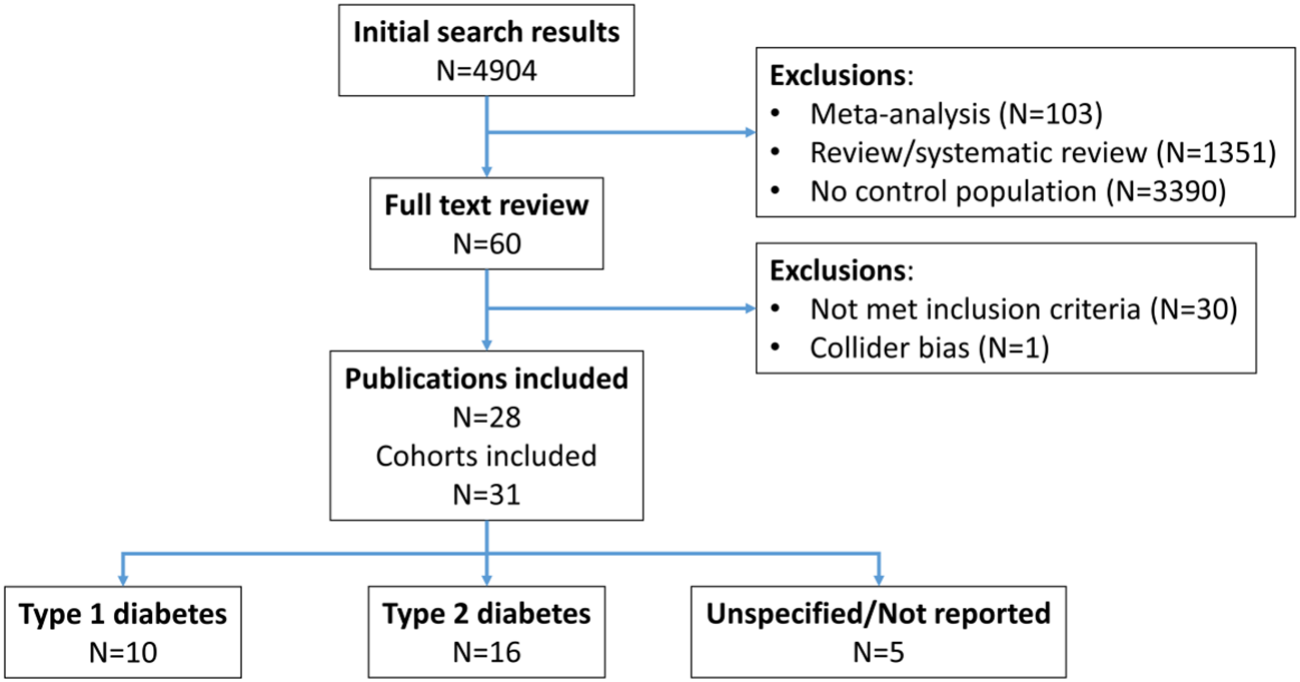
Flow chart of the selection of included studies.

The flow chart of the search strategy is presented in **Figure 1**. An initial search produced 4904 articles (**Figure 1**). Based on titles and abstracts, we excluded 4844 articles that reported no original data that didn’t investigate DSPN or didn’t report mortality data, or had no control population (without DSPN), leaving 60 papers for full text retrieval. All these papers were written in English. We excluded a further 31 papers due to different reasons leaving 29 papers that could be included in the meta-analysis. Of the 29, two studies (17, 37) reported estimates separately for type 1 and type 2 diabetes and one (14) reported estimates separately for two population-based cohorts (ADDITION and DD2) that allowed us to use them as altogether six separate cohorts. In contrast, we excluded 2 studies from the unadjusted analysis (37, 44), due to the potential of collider bias. Our final sample for the main analysis thus included 31 cohorts and for the fully adjusted analysis 17 cohorts (12, 14–40).

### Characteristics of included studies

Detailed characteristics of the included cohorts are summarized in **Table 1**. Out of the included 31 cohorts, 10 reported on type 1 diabetic patients, 16 on type 2 diabetic patients. In the remaining 6 papers, type of diabetes was either not reported, or estimates could not be separated for patients with type 1 and type 2 diabetes.

Nine of the cohorts included population-based samples, while most were performed in tertiary care (14 in single, 2 in multiple centers). Six cohorts used data from randomized trials or their follow-up cohorts (**Table 1**).

Diagnosis of DSPN varied widely between the different cohorts. Five cohorts used medical records as the source of data, leading to potential bias related to the non-standardized ways of examination. 3 cohorts used non-standardized symptom-checks only for diagnosis, 6 cohorts standardized questionnaires, 8 physical examination, and 7 studies used different equipments that provide quantitative measures of neuronal function. Only 5 studies used a combination of the above methods to provide the diagnosis of DSPN. When quantitative or semi-quantitative methods were considered as more reliable tools for the diagnosis of DSPN, 18 studies fulfilled this requirement (**Table 1**).

Altogether over 150 thousand diabetes patients (54% male) were included in the main analysis of whom 12.3% died during the median 7.5 (range 1.9-15.0) years of follow-up. The median proportion of participants with DSPN was 27.4% (range 8.8%-73.6%).The median age of participants was 60 years (range 20.8-68.0 years), diabetes duration ranged 0 to 25 years (median 11.1 years) in the different cohorts (**Table 1**).

While all studies provided some estimates of mortality, unadjusted (including those only adjusted for age and sex) estimates were available for 29 cohorts, while multiple adjusted estimates were reported in 17 cohorts (**Table 1**).

According to the NOS classification, 18 studies had good, 1 study had fair, and 9 poor quality (**Supplementary Table S1**).

### Association between DSPN and mortality

**Figure 2:**
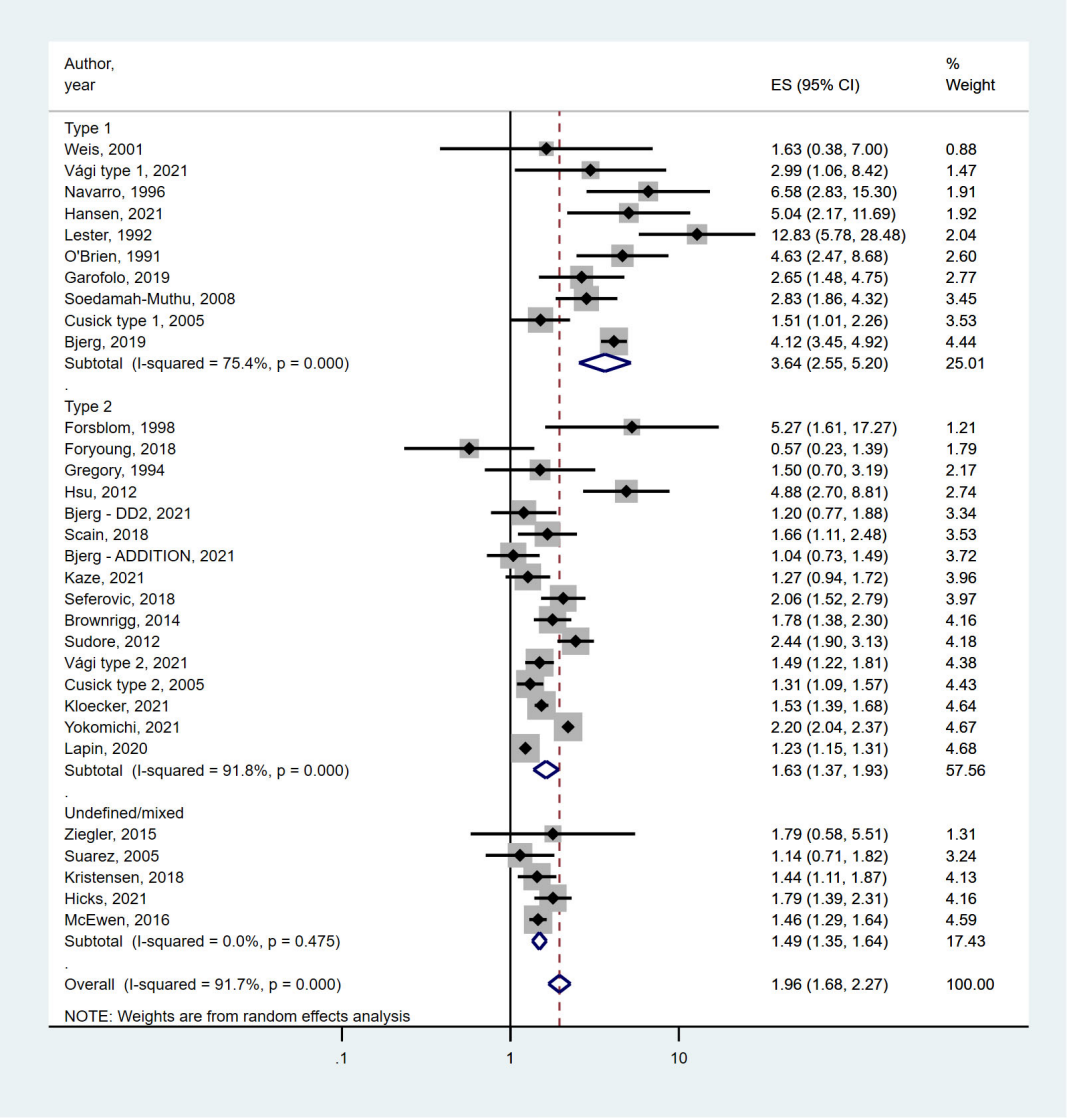
Forest plot and pooled estimates of the association between DSPN and all-cause mortality stratified by type of diabetes. Error bars show 95% confidence intervals. *Abbreviations: ADDITION*: Anglo-Danish-Dutch Study of Intensive Treatment in People With Screen-Detected Diabetes in Primary Care; *CI*: confidence interval; *DD2*: Danish Centre for Strategic Research in Type 2 Diabetes; *ES*: effect size;.

In the pooled primary analysis, the presence of DSPN at baseline was significantly associated with an almost doubled risk of mortality (pooled HR 1.96, 95%CI 1.68-2.27). While the point estimates in the individual studies were over one for all but one studies, the heterogeneity between studies was large (I^2^ = 91.7%, p<0.001) (**Figure 2**).

According to our primary hypothesis we stratified the analysis between DSPN and mortality by the type of diabetes. Among patients with type 1 diabetes, the mortality was 3.64 (95%CI 2.55-5.20) times higher in the presence of DSPN compared to those without DSPN. While the I^2^ value (75.4%, p<0.0001) was somewhat lower in these studies compared to the overall value, heterogeneity remained large (**Figure 2**).

The pooled association between DSPN and mortality was weaker in patients with type 2 diabetes (pooled HR 1.63, 95%CI 1.37-1.93), although DSPN still was a strong predictor of all-cause mortality. There was large heterogeneity in the estimates between studies reflected by the high I^2^ value (91.8%, p<0.0001), partly related to the study by Foryoung et al. that reported a decreased risk of mortality in patients with DSPN (**Figure 2**).

In cohorts where it was impossible to assess the risks by diabetes type, we found a similar association between DPSN and mortality as in type 2 diabetes (pooled HR 1.49, 95% CI 1.35-1.64). There was a low heterogeneity between the individual risk estimates (I^2^ 0%, p=0.48), suggesting that these estimates may have come from a common background population (probably mostly type 2 diabetes patients) (**Figure 2**).

When we formally tested for a difference in the estimates between type 1 and type 2 diabetes by meta-regression, we found that the risk associated with DSPN was more than 2 times higher in type 1 compared to type 2 diabetes (HR 2.22, 95%CI 1.43-3.45, p=0.001).

We also investigated the potential for publication bias in our estimates. Given the large difference in risks between diabetes types, we drew funnel plots separately for each type of diabetes. These funnel plots confirmed the large difference between type 1 and type 2 (and also undefined diabetes) groups but were incompatible with the presence of strong publication bias. The Egger tests were all non-significant (all p>0.8) further confirming the visual interpretation (**Supplementary Figure 2**).

We also tested for influential studies by eliminating individual studies from the meta-analysis run for each type of diabetes. All these analyses showed overlapping confidence intervals with the overall (diabetes type specific) estimates arguing against the role of some influential studies in the findings. (Figures are available on request.)

### Sensitivity analyses

Our pre-planned sensitivity analyses restricted included cohorts by level of covariate adjustment, type of study setting, diagnostic test used to define DSPN, and overall quality of studies.

First, we run separate analyses including 29 studies that reported unadjusted, and 17 studies that reported adjusted estimates. The overall risk associated with DSPN was smaller according to the adjusted estimates (pooled HR 2.0 vs. 1.6) suggesting that the included covariates explained some of the increased mortality in DSPN. In contrast, the difference between type 1 and type 2 diabetes remained statistically significant showing stronger associations between DSPN and mortality in type 1 compared to type 2 diabetes (**Table 2**).

**Table 2:** Association between DSPN and all-cause mortality in sensitivity analyses restricted to cohorts based on adjustment of estimates, study setting, measurement methods, NOS score, and those that reported diabetes duration.

Type of studies included	All studies			Type 1 diabetes			Type 2 diabetes			p
	N	HR (95% CI)	I^2^	n	HR (95% CI)	I^2^	n	HR (95% CI)	I^2^	
**Unadjusted analyses**	29	2 (1.7-2.35)	92.5%	9	3.95 (2.73-5.73)	77.6%	15	1.64 (1.37-2.35)	92.2%	0.001
**Adjusted analyses**	17	1.6 (1.37-1.87)	78.8%	4	2.36 (1.52-3.66)	73.5%	11	1.46 (1.25-1.7)	67.3%	0.026
**Population-based studies**	8	1.83 (1.36-2.47)	80.3%	0	NA	NA	6	2.03 (1.35-3.05)	84.1%	NA
**Quantitative or semi-quantitative tests**	18	2.36 (1.82-3.06)	87.7%	8	3.31 (2.35-4.65)	72.5%	7	1.82 (1.41-2.37)	74.2%	0.046
**Good NOS score**	16	1.80 (1.51-2.14)	83.3%	4	2.65 (1.59-4.42)	70.0%	10	1.67 (1.33-2.10)	87.5%	0.164
Reported data on diabetes duration*	22	2.23 (1.72-2.86)	NA	9	3.39 (1.93-5.93)	NA	13	1.65 (1.16-2.32)	NA	0.077

P is given for the meta-regression estimate investigating the difference of estimates between type 1 and type 2 diabetes.

* Based on a meta-regression with type of diabetes and duration of diabetes as covariates. I^2^ cannot be calculated from meta-regression.

CI, confidence interval; HR, hazard ratio; NOS, Newcastle-Ottawa Scale.

NA, Not applicable.

When we restricted the analysis to population-based studies, no cohort performed on type 1 diabetes patients remained. The overall risk estimates however confirmed the association between DSPN and mortality with an almost 2 times higher risk in patients with DSPN (**Table 2**).

When we restricted the analysis to cohorts that used quantitative or semi-quantitative methods to define DSPN, the overall association between DSPN and mortality remained and the point estimate was similar to those of the other analyses. Furthermore, heterogeneity was somewhat smaller for both types of diabetes (<75%), suggesting that the use of different methods could increase heterogeneity in the mortality estimates. Although the absolute difference in hazard ratios between type 1 and type 2 diabetes was smaller in this analysis, the estimates remained statistically significantly different further supporting our hypothesis that a stronger association exists between DSPN and mortality in type 1 diabetes (**Table 2**).

When we restricted our analysis to cohorts that deemed to be of good quality according to the NOS scale, we found point estimates similar to those from the main analysis, confirming the association between DSPN and mortality. Although the difference between type 1 and type 2 diabetes pointed to the same direction as in the main analysis, the difference between hazard ratios was statistically not different probably due to the limited number of studies included in this analysis (**Table 2**).

Finally, in a meta-regression adjusted for diabetes duration, we found a similarly increased mortality in type 1 diabetes compared to type 2 diabetes as in the main analysis, although due to the wide confidence intervals, it lost its statistical significance.

## Discussion

### Short summary

Based on a meta-analysis of 31 cohorts comprising over 150 thousand diabetic patients, we found an almost doubling (HR 1.96, 95%CI 1.68-2.27) of all-cause mortality among diabetes patients with distal symmetric polyneuropathy compared to those without DSPN. The association was somewhat attenuated but still statistically and clinically significant (HR 1.60, 95%CI 1.37-1.87) in those studies that take into account multiple risk factors of vascular disease and all-cause mortality. The observed association was more than two times stronger in type 1 compared to type 2 diabetes (HR 2.22, 95%CI 1.43-3.45), most likely reflecting the much smaller background mortality of younger patients without DSPN. The observed findings were robust in several sensitivity analyses that aimed to remove bias related to different study designs, methods for DSPN definitions, and overall study quality. Furthermore, we found no sign of publication bias, or any individual study with a major influence on the estimates.

### Findings in context

#### Increased risk of mortality in DSPN

Our overall finding of a statistically significantly increased mortality among people with diabetes is not very surprising given the fact that more than two third (22/31) of the included cohorts found a significantly increased risk of mortality and a further 8 studies had point estimates above one. It should be noted however, that only 4 cohorts aimed at specifically investigating the association between DSPN and all-cause mortality (12, 23, 37). While two of these showed similarly increased mortality to the overall estimate (12, 37), the other two showed substantially higher risks (although one cohort included only type 1 diabetes patients) (23, 37).

Most of the other cohorts that reported adjusted estimates for the DSPN — all-cause mortality association, aimed to investigate either microvascular complications (or just risk factors) as predictors or cardiovascular outcomes according to their primary hypothesis. Some of the cohorts reported only unadjusted estimates that is related to the fact that these cohorts were aiming at investigating completely different questions, although reported on crude frequencies of DSPN and mortality.

#### Role of other risk factors of mortality

The fact that the association between CAN and all-cause mortality was found to be substantially attenuated and even became non-significant after adjustment for known hypertension or nephropathy in the Pittsburgh Epidemiology of Diabetes Complications (EDC) Study (45) highlights the importance of taking into account estimated risk of mortality of the participants.

Given the above reasoning, we preplanned to analyze cohorts separately that reported estimates on the DSPN-mortality association using multiple adjustments for risk factors in addition to age and sex. The role of these confounders is obvious, given that in addition to glycemic control, other cardiovascular risk factors (for example, hyperlipidemia, hypertension, abdominal obesity, smoking, alcohol use) and micro- and macrovascular complications of diabetes are strong predictors of DSPN (8, 46, 47).

Altogether 55% (17/31) cohorts reported adjusted estimates that confirmed our hypothesis that the association would be attenuated compared to findings of the main analysis. It should be noted however that the association still remained strong with a 60% increased point estimate (HR 1.6, 95%CI 1.37-1.87) and a confidence interval that also suggests an important effect size.

#### Differences between T1DM and T2DM

Although 7 studies included both type 1 and type 2 diabetes patients, only 2 of these (17, 37) reported estimates separately for type 1 and type 2 diabetes reporting equivocal findings. Vági et al. showed increased risk in type 1 diabetes, while Cusick reported similar risks in type 1 and type 2 diabetes. Our meta-analysis strongly supports the hypothesis that the mortality risk is much stronger in type 1 than in type 2 diabetes with a more than 2 times increased risk in type 1 vs type 2 diabetes (HR 2.22, 95%CI 1.43-3.45). An indirect support of the validity for the risk difference between type 1 and type 2 diabetes comes from a meta-analysis that reported similar observation between CAN and all-cause mortality (7).

We suspect that this observation is mostly related to the huge age difference between type 1 and type 2 diabetes patients. Younger diabetes patients without DSPN are likely to be free of diabetes complications (given the strong grouping of these complications) and have a good general health, while those with DSPN are likely to have other complications, hence a hugely increased mortality. In contrast older patients with type 2 diabetes have several vascular risk factors (such as obesity, hypertension, hyperlipidemia) and an elevated mortality already independent of DSPN status leading to a smaller relative mortality increase. However it is important to note here that given the much lower absolute risk in younger people, the absolute risks still remain much higher in type 2 compared to type 1 diabetes (15, 48). It is also possible that DSPN has different etiologies in type 1 and 2 diabetes with mostly related to hyperglycemia in type 1 diabetes vs a multifactorial (partly non-glycemia related) origin in type 2 diabetes (12). A potential further explanation could be related to the fact that adults with type 1 diabetes usually have more severe disease related to longer diabetes duration and worse glycemic control. While our sensitivity analysis adjusted for diabetes duration argues against the role of diabetes duration, we could not test the role of long-term glycemia due to poor quality data on glycemic control.

It should be noted that the relative mortality difference between type 1 and type 2 diabetes became non-significant in some of the sensitivity analyses. Given that the point estimates even in those models remained well above unity (1.6-2.0), we suspect that this is most likely a power issue.

#### Role of study design

In contrast to our hypothesis, study design had no strong effect on the association of DSPN with all-cause mortality supported by the similar overall estimates in our main analysis and in a sensitivity analysis restricted to population-based studies. Furthermore, we found no influential study that would significantly alter the findings. We excluded one study at the selection phase based on the possibility of referral bias (44). This study reported unadjusted association of DSPN with mortality in a population that was referred with symptoms to a neuropathy center. For the same reason, we excluded unadjusted estimates from the study of Vági et al., however this study also reported multiple adjusted estimates that seemed to control for the imbalance in mortality risk factors between patients with or without DSPN (37).

#### Role of DSPN measurement methods

The meta-analyses that evaluated the effect of diabetes related CAN with all-cause mortality suggested that the method used for the assessment of neuropathy has a strong effect on the association with larger estimates if more than one modality was taken into account (6, 7). Based on this observation, we preplanned a sensitivity analysis restricted to those studies that used quantitative or semiquantitative tests for diagnosis.

The definition of DSPN in the different studies varied widely. Some of the cohorts used administrative data for the definition of DSPN that is prone to (for example) indication bias, we defined these measures as high risk of bias. Another, unstandardized way for the definition is the use of simple questions on the symptoms of DSPN. Given that their wording is different between studies, these could introduce variability when meta-analyzed. Simple physical examination of absent reflexes by themselves are weak instruments for the diagnosis of DSPN and they are becoming less frequently used in research partly due to their subjectivity.

A potential way to improve the symptom-based definition of DSPN is the use of standardized questionnaires. Several studies used the validated Michigan Neuropathy Screening Instrument (MNSI) for the screening of DSPN (49). It should be mentioned however that it has a low sensitivity (26-40%) with potential differences between type 1 and type 2 diabetes patients (50, 51). Beside MNSI, other, less validated questionnaires were also used in some studies. The low sensitivity of the questionnaire methods could underestimate the association of DSPN with all-cause mortality.

We selected studies that used different instruments for the definition of DSPN as the most reliable tests. While the used tests are heterogeneous, given their quantitative nature and the use of equipment, they are in general less subjective. Furthermore, a skilled assistant could complete the investigation, making them suitable for the testing of large populations. These methods are also advocated in clinical guidelines for the screening of DSPN. However, it should be noted that these tests measure different modalities of sensation (i.e. pain, touch, vibration, electric current) (52, 53).

A potential way to improve the definition of DSPN would be the use of a combination of a standardized questionnaire and equipment-based methods. However the number of studies that used both of these are so limited that we were unable to perform a meta-analysis with this definition (**Table 1**).

Our finding that the association was stronger in studies that used semiquantitative methods for the definition of DSPN suggests that the risk associated with DSPN is probably underestimated in our main analysis. Furthermore, this sensitivity analysis is also compatible with a higher relative mortality in type 1 vs type 2 diabetes with DSPN.

#### Role of overall study quality

We used the Newcastle-Ottawa scale to measure the overall quality of studies that includes all the above detailed aspects of study design.(NOS) The findings of the analysis restricted to good quality studies confirmed our findings in the main analysis with mostly overlapping confidence intervals (5, 41).

### Potential mechanisms

The mechanism responsible for the association between DSPN and mortality is not well investigated. In general, DSPN is thought to be a determinant of reduced quality of life through disturbed sleep, physical functioning, recreation, and diminished physical and emotional well-being (54). However, the neuroendocrine, proinflammatory, and neurodegenerative underpinnings of DSPN could also lead to cardiovascular disease through increased oxidative stress and levels of advanced glycation end products (55, 56). DSPN is also associated with balance impairment that could lead to falls and injuries (56). DSPN is a leading factor of diabetic foot ulcers and amputations, both associated with increased mortality through infection and chronic inflammation (57).

Furthermore, diabetic pain per se (through similar mechanisms as DSPN itself) could be associated with an even further increased risk of mortality, as suggested by the observation of Lapin et al. on increased mortality only in those patients with painful DSPN but not in those with DSPN without pain compared to DSPN free controls (27). This hypothesis seems to be further strengthened by a meta-analysis that suggests an increased risk of mortality in people with widespread body pain irrespective of its origin even after adjustment for some mortality risk factors (58).

Alternatively, it is also possible that DSPN is a marker of other diseases that increase mortality. Indeed, microvascular diabetes complications (especially DSPN and CAN) show remarkable clustering (59). Thus, it is possible that the association between DSPN and mortality could be mediated through CAN.

### Strengths and limitations

Our meta-analysis was based on a predefined protocol standardizing the inclusion of studies and also the testing of our hypotheses. Overall, our current analysis is based on a sample with over 150 thousand participants with sufficiently high rates of mortality and DSPN to provide stable estimates. The main outcome (all-cause mortality) is a hard outcome that is unlikely to be imprecisely reported. The observed effect size is clinically relevant and statistically significant. The similar results in our main and sensitivity analyses further confirm the observed strong associations. Furthermore, our tests for influential studies and publication bias argues against major effects of individual studies or selective publication.

As the quality of any meta-analysis is mostly determined by the quality of the included studies, the main limitations of our report are mostly related to the quality of the included studies. First, a large proportion of the included studies include patients from tertiary care centers, from high-income countries with Caucasian origin. All of these factors limit the external validity of our findings. Furthermore, although some of the studies reported on multiple adjusted associations between DSPN and all-cause mortality, the role of unmeasured confounding cannot be excluded. The included studies had limited data on important mortality risk factors, such as different medications, laboratory parameters, proportion of participants with risk factors at target, or other comorbidities with increased mortality. Furthermore, the differences in the definition of DSPN could have biased our results. Given that we included only cohort studies, bias related to lost to follow-up cannot be excluded. While all-cause mortality is an easily obtainable outcome, it would be of interest to see the associations of DSPN with different causes of death.

## Conclusion

Our meta-analysis strongly suggests that distal symmetric neuropathy in diabetes patients is associated with a substantially increased all-cause mortality in both type 1 and type 2 diabetes. Even if this finding is not causal, this observation should have an effect on clinical practice: the known modifiable risk factors of mortality should be treated more stringently in the presence of DSPN similarly to people with type 2 diabetes and chronic kidney disease (60). If this finding is causal, the lack of an etiological treatment of DSPN becomes even more important. The finding of a stronger association between DSPN and mortality in type 1 diabetes highlights the fact that the deleterious effect of DSPN is not limited to older people with type 2 diabetes.

## Data availability statement

The original contributions presented in the study are included in the article/**Supplementary Material**. Further inquiries can be directed to the corresponding author.

## Author contributions

AT and MS contributed to the study conception and design. Analysis and interpretation were handled by all authors. Drafting of the article was handled by OV, MS, BD, VH, and AT. Critical revision for intellectual content was handled by all authors. All authors contributed to the article and approved the submitted version.
