# Supplementary material for: The association between distal symmetric polyneuropathy in diabetes with all-cause mortality – a meta-analysis
Source: Front Endocrinol (Lausanne). 2023 Feb 16;14:1079009. doi: 10.3389/fendo.2023.1079009 (PMC9978416; doi:10.3389/fendo.2023.1079009)
Supplement: Supplementary file 1 [file DataSheet_1.pdf]

Supplementary tables and figures

Supplementary Table 1. Newcastle-Ottawa quality assessment scale (NOS) for observational studies included in the meta-analysis.

Author, year	Selection	Comparability	Exposure/Outcome	Overall
Forsblom, 1998	4	2	3	9
Hicks, 2021	4	2	3	9
Hsu, 2012	4	2	3	9
Scain, 2018	4	2	3	9
Vági, 2021	4	2	3	9
Brownrigg, 2014	4	2	2	8
Cusick, 2005	4	2	2	8
Kaze, 2021	3	2	3	8
O'Brien, 1991	4	2	2	8
Soedamah-Muthu, 2008	4	2	2	8
Yokomichi, 2021	3	2	3	8
Bjerg, 2019	4	0	3	7
Bjerg, 2021	3	2	2	7
Hansen, 2021	4	0	3	7
Kristensen, 2018	3	2	2	7
McEwen, 2016	4	0	3	7
Suarez, 2005	4	0	3	7
Ziegler, 2015	4	0	3	7
Seferovic, 2018	2	2	3	7
Gregory, 1994	3	0	3	6
Kloecker, 2021	3	0	3	6
Lapin, 2020	3	0	3	6
Garofolo, 2019	2	0	3	5
Lester, 1992	3	0	2	5
Navarro, 1996	2	0	3	5
Weis, 2001	2	0	3	5
Sudore, 2012	1	0	2	3
Foryoung, 2018	0	0	2	2

Thresholds for converting the Newcastle-Ottawa Scales to AHRQ standards (good, fair, and poor): Good quality: 3 or 4 stars in selection domain AND 1 or 2 stars in comparability domain AND 2 or 3 stars in outcome/exposure domain Fair quality: 2 stars in selection domain AND 1 or 2 stars in comparability domain AND 2 or 3 stars in outcome/exposure domain Poor quality: 0 or 1 star in selection domain OR 0 stars in comparability domain OR 0 or 1 stars in outcome/exposure domain.

Supplementary Figure 1. Funnel-plot of standard error by log-hazard ratio (HR) for studies examining the association between DSPN and all-cause mortality for studies in type 1 diabetes (A), type 2 diabetes (B), and mixed/undefined types of diabetes (C).

A

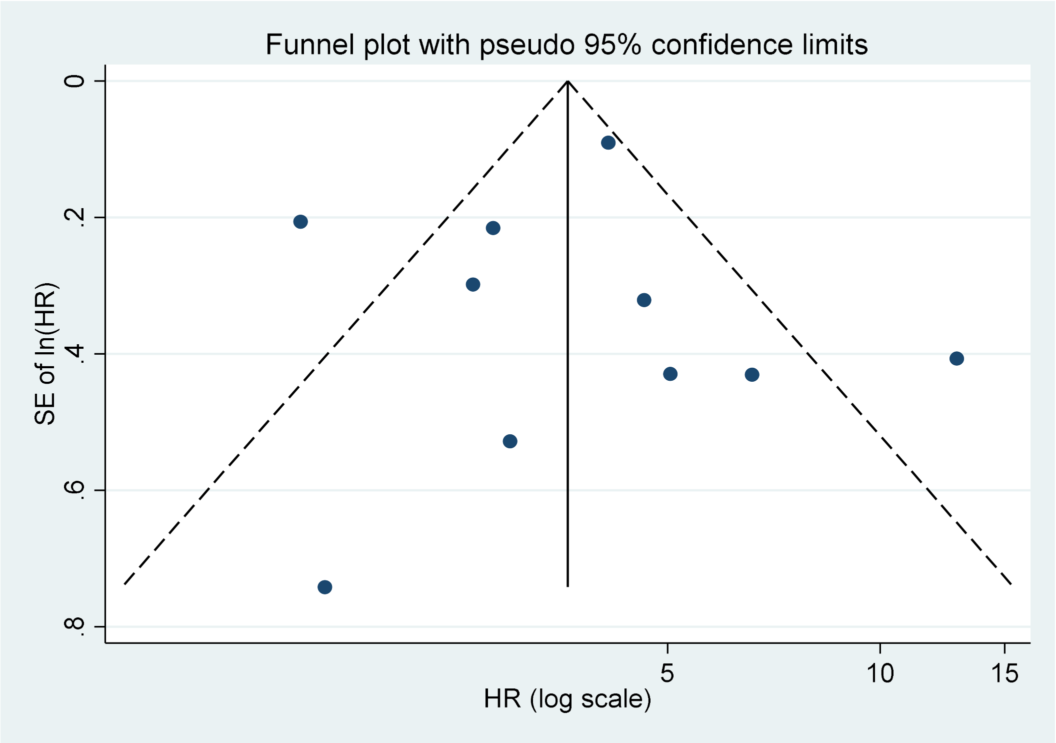

B

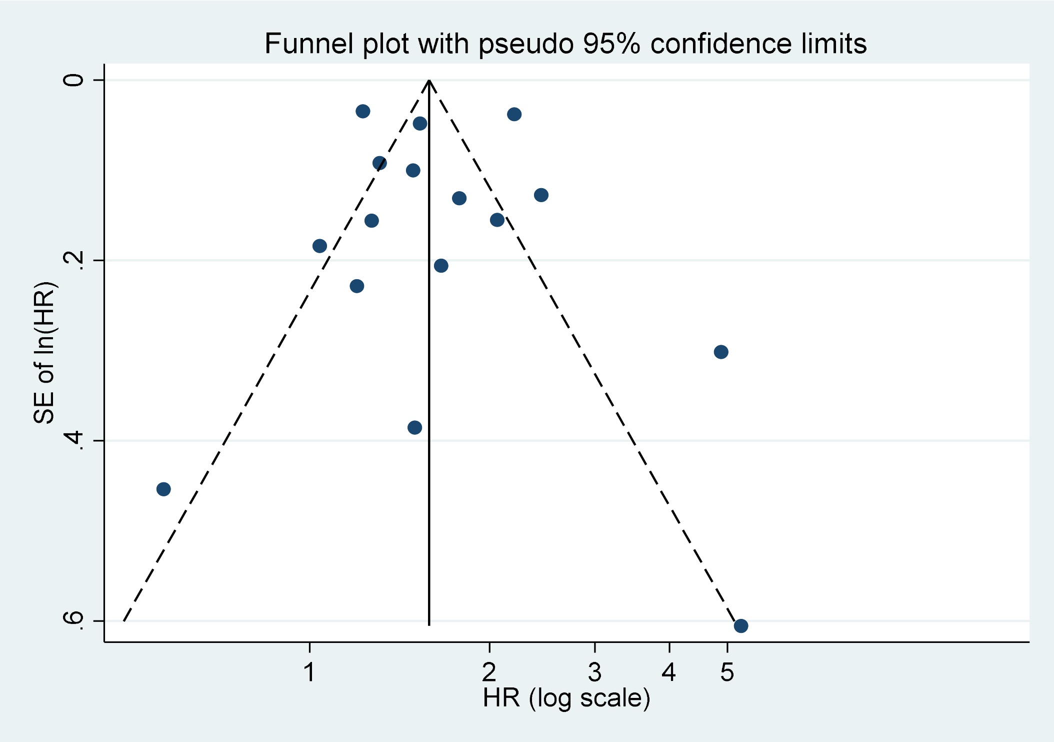
Funnel plot with pseudo 95% confidence limits
SE of ln(HR)
0
.2
.4
.6
1
2
3
4
5
HR (log scale)

C

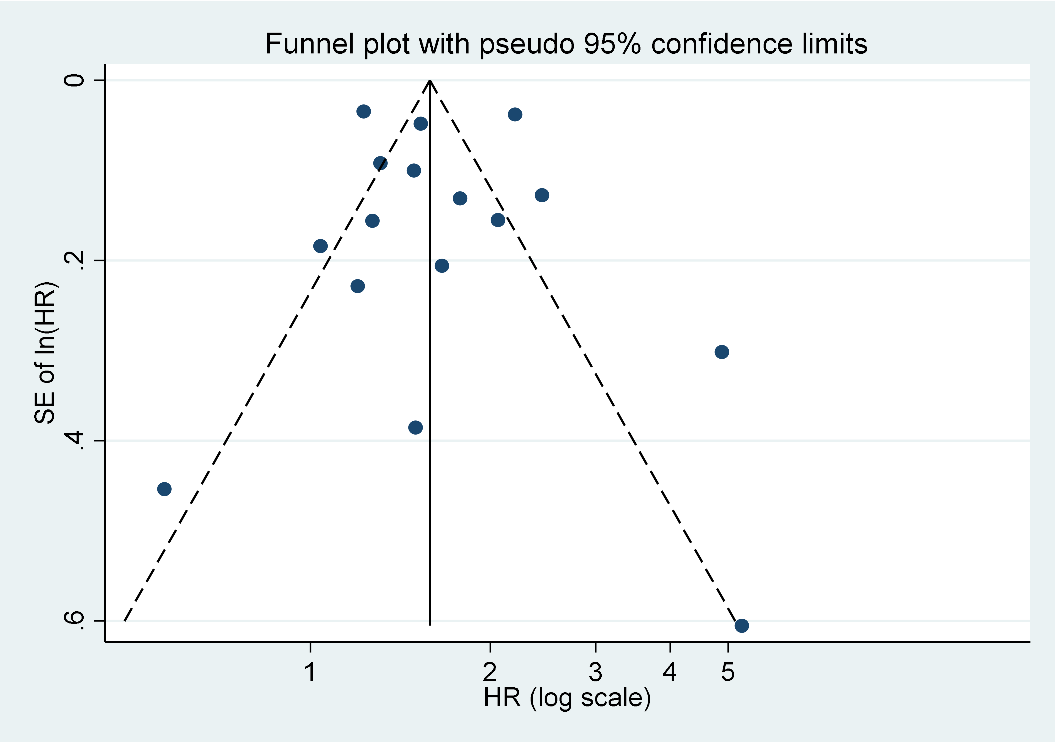

P values for Egger tests: type 1 diabetes p=0.943, type 2 diabetes p=0.801, mixed/undefined p=0.95.

Abbreviations: *DSPN*: distal symmetric polyneuropathy; *HR*: hazard ratio
